# The traditional Chinese formulae Ling-gui-zhu-gan decoction alleviated non-alcoholic fatty liver disease via inhibiting PPP1R3C mediated molecules

**DOI:** 10.1186/s12906-018-2424-1

**Published:** 2019-01-07

**Authors:** Yanqi Dang, Shijun Hao, Wenjun Zhou, Li Zhang, Guang Ji

**Affiliations:** 1 Institute of Digestive Diseases, Longhua Hospital, Shanghai University of Traditional Chinese Medicine, Shanghai, 200032 China; 2 Zhoupu Hospital, Shanghai University of Medicine &Health Sciences, Shanghai, 201318 China

**Keywords:** Ling-gui-zhu-Gan decoction, Non-alcoholic fatty liver disease, PPP1R3C, Glycogen metabolism, Lipogenesis

## Abstract

**Background:**

Ling-gui-zhu-gan decoction (LGZG), a classic traditional Chinese medicine formula, has been confirmed to be effective in improving steatosis in non-alcoholic fatty liver disease (NAFLD). However, the mechanism under the efficacy remains unclear. Hence, this study was designed to investigate the mechanisms of LGZG on alleviating steatosis.

**Methods:**

Twenty four rats were randomly divided into three groups: normal group, NAFLD group, fed with high fat diet (HFD) and LGZG group (fed with HFD and supplemented with LGZG). After 4 weeks intervention, blood and liver were collected. Liver steatosis was detected by Oil Red O staining, and blood lipids were biochemically determined. Whole genome genes were detected by RNA-Seq and the significant different genes were verified by RT-qPCR. The protein expression of Protein phosphatase 1 regulatory subunit 3C (PPP1R3C) and key molecules of glycogen and lipid metabolism were measured by western blot. Chromophore substrate methods measured glycogen phosphorylase (GPa) activity and glycogen content.

**Results:**

HFD can markedly induce hepatic steatosis and promote liver triglyceride (TG) and serum cholesterol (CHOL) contents, while liver TG and serum CHOL were both markedly decreased by LGZG treatment for 4 weeks. By RNA sequencing, we found that NAFLD rats showed significantly increase of PPP1R3C expression and LGZG reduced its expression. RT-qPCR and Western blot both verified the alteration of PPP1R3C upon LGZG intervention. LGZG also promoted the activity of glycogen phosphorylase liver type (PYGL) and inhibited the activity of glycogen synthase (GS) in NAFLD rats, resulting in glycogenolysis increase and glycogen synthesis decrease in the liver. By detecting glycogen content, we also found that LGZG reduced hepatic glycogen in NAFLD rats. In addition, we analyzed the key molecules in hepatic de novo lipogenesis and cholesterol synthesis, and indicated that LGZG markedly inhibited the activity of acetyl-CoA carboxylase (ACC), sterol receptor element-binding protein-1c (SREBP-1c) and 3-hydroxy-3-methylglutaryl-CoA reductase (HMGCR), resulting in lipid synthesis decrease in the liver.

**Conclusion:**

Our data highlighted the role of PPP1R3C targeting pathways, and found that hepatic glycogen metabolism might be the potential target of LGZG in preventing NAFLD.

**Electronic supplementary material:**

The online version of this article (10.1186/s12906-018-2424-1) contains supplementary material, which is available to authorized users.

## Background

Non-alcoholic fatty liver disease (NAFLD) is the hepatic manifestation of metabolic syndrome, and featured as accumulation of fat deposits in the liver [1, 2]. Importantly, NAFLD is one of the most dangerous liver complications, such as fibrosis, cirrhosis and hepatocellular carcinoma [3, 4]. Furthermore, accumulating evidences indicate that NAFLD can be associated with a series of chronic diseases, such as cardiovascular disease, chronic kidney disease and type 2 diabetes mellitus [5, 6], making NAFLD a major public health concern worldwide [7].

NAFLD can affect glycogen metabolism [8], which is the primary storage form of excess energy. Protein phosphatase 1 regulatory subunit 3C (PPP1R3C) is an enzyme that binds to protein phosphatase-1 (PP1) as a regulator which can mediate glycogen metabolism [9]. PPP1R3C encoded protein is called protein targeting to glycogen (PTG) [10]. PTG overexpression can increase glycogen storage [11]. PTG knocked-down could suppress the cellular glycogen level in mice, and heterozygous deletion of PTG in mice also showed glucose and insulin resistance [12, 13]. Glycogen synthase kinase 3β (GSK3β), glycogen synthase (GS) and glycogen phosphorylase liver type (PYGL) are the down-steam targets of PTG [14, 15]. PTG enhances the de-phosphorylation of GS and causes the activation of glycogen synthesis [16–18]. GSK3β also takes part in regulating the phosphorylation of GS. PTG is reported to inhibit PYGL expression and phosphorylase (GPa) de-phosphorylation [16, 17], the phosphorylated form of GPa is catalytically active and catalyzes glycogenolysis in liver.

Up to now, therapeutic strategies for NAFLD are very limited, thus exploring proper agents in preventing NAFLD are urgent. Traditional Chinese medicine (TCM) has long been practiced in clinic, and several formulas are confirmed to be effective in treating NAFLD [19–22]. Ling-gui-zhu-gan decoction (LGZG) is an ancient formula derived from the classic work of TCM titled Jin-Gui-Yao-Lue. Recently, LGZG was found to be effective in metabolic syndrome, e.g. obesity, hyperglycemia, hyperlipidemia, hypertension. Furthermore, animal studies identified LGZG could improve dyslipidemia and decrease inflammatory cytokines in hyperlipidemia rats [21, 23]. And our previous work showed that LGZG could attenuate induced NAFLD in high fat diet (HFD) feeding rats [24, 25].

Although the effect of LGZG has been confirmed, the mechanisms under the efficacy are rather elusive. In the present study, we applied HFD induced NAFLD rats to evaluate the efficacy of LGZG [26]. Based on RNA-Sequence data, we focused on the PTG function and its related regulation on liver glycogen metabolism, trying to clarify the potential mechanisms of LGZG in preventing NAFLD.

## Methods

### Preparation of Ling-gui-zhu-Gan decoction

Ling-gui-zhu-gan decoction comprises: *Poria* (voucher No. 160220), *Ramulus Cinnamomi* (voucher No. 160702), *Rhizoma Atractylodis Macrocephalae* (voucher No. 160311), and *Radix Glycyrrhizae* (voucher No. 160215). Voucher specimen of each species was deposited at Longhua Hospital affiliated to Shanghai University of TCM. The ratio of the four herbs was 2:1.5:1.5:1, all herbs were provided by Longhua Hospital affiliated to Shanghai University of TCM. TCM pharmacologist Tong Zhang undertook the formal identification of the four plant materials and provided the fingerprint spectrum by LC-MS (Additional file 1: Figure S1). Herbal decoction was prepared as previously described [24]. Briefly, (1) Mixed the herbal materials in a cooking pot with 500 mL water; (2) boiled the mixture for 30 min; (3) simmered for another 20 min; (4) transferred the liquid by filtration. The final concentrated decoction was 100 ml.

### Animals and diets

Five- week- old male Wistar rats (130 g ± 10 g) were obtained from Shanghai SLAC Laboratory Animal Co. Ltd., China, and maintained under a controlled temperature (23 ± 3 °C) and humidity (55 ± 15%) with a 12 h light/12 h dark cycle for 1 week. After acclimation, the 24 rats were randomly divided into three groups: normal group (*n* = 8), fed with chow diet; NAFLD group (*n* = 8), fed with HFD (88% chow diet, 10% lard and 2% cholesterol); LGZG group (*n* = 8), fed with HFD and supplemented with LGZG (10 ml/kg/d) for 4 weeks via gavage. At the end of experiment, animals were weighed and injected 2% pentobarbital sodium (3 ml/kg body weight) for anesthesia. Blood was collected and serum was separated for biological analysis, liver tissues were quickly removed, rinsed with 0.9% sodium chloride solution and then sacrificed the animals via exsanguination. Liver tissues were weighed and stored in liquid nitrogen. All the animals received humane care according to the Chinese Animal Protection Act and National Research Council criteria and animal ethic (PZSHUTCM18101801) was approved by the Animal Experiment Ethics Committee of Shanghai University of Traditional Chinese Medicine.

### Histopathological examination

Frozen liver tissues (central part of the left lateral lobe) were placed in optimal cutting temperature compound, cut into 8 μm sections and stained with Oil-Red O solution. Images were taken under Olympus IX71 Inverted microscope (Tokyo, Japan) at 200 × magnification.

### Biochemical analysis

Serum triglyceride (TG), cholesterol (CHOL) and non-esterified fatty acid (NEFA) were analyzed using the Hitachi full-automatic system with corresponding kits (Wako, Richmond, VA, USA). Liver TG was assayed using commercial kits (Nanjing Jiancheng Bioengineering Institute, Nanjing, China) according to the manufacturers’ instruction.

### RNA-seq detection and data analysis

All liver samples for RNA extraction were from the central part of the right inferior lobe, and total RNA was extracted (7 samples per group) using Trizol reagent (Invitrogen, USA). cDNA libraries were established using KAPA Stranded RNA-Seq Library Prep Kit (Illumina, USA) according to the manufacturer’s instructions. Quality of libraries was verified by Agilent 2100 Bioanalyzer, quantified and sequenced on the Illumina HiSeq 4000 according to the standard sequencing protocol.

### Quantitative real-time polymerase chain reaction

Real-time quantity polymerase chain reaction (RT-qPCR) amplification and detection were performed using the SYBR Green PCR Mix (Life Technologies) in a StepOne real-time PCR system (Life Technologies) according to the manufacturer’s protocol. Gene expression was normalized using β-actin as a reference gene. The primers were listed in Table 1.

**Table 1:** The primers sequence involved in the paper

gene	forward	reverse
PPP1R3C	5’-ATTTGCTTGGCACATTCACC-3’	5′- GTGGGCTCTTCCATTCCTTC-3’
SLC38A2	5’-CAGTTGGGACATAAGGCATACG-3’	5’-ATAGTCGCCGTTCAGATACCAC-3’
OSGIN1	5′- GCAGCAGATGATGCGTGAC -3’	5′- GGAGCCGATGAGGACGAG − 3’
ZFP189	5′- GAAAGACATCGAACCACAGGG − 3’	5’-TGTTCCTCAGTCAAAGAATCACG-3’
β-actin	5’-CCCATCTATGAGGGTTACGC-3’	5’-TTTAATGTCACGCACGATTTC-3’

### Western blot

50 mg liver tissue was lysed in RIPA lysis buffer and centrifuged for 10 min at 12000 g. The supernatant was collected for protein concentration measurement using protein assay kit (BioRad, Hercules, CA, USA), and 30 μg of protein was separated through sodium dodecyl sulfate-polyacrylamide gel electrophoresis and transferred to nitrocellulose membranes (Merck Millipore, USA). The membranes were blocked overnight with 5% non-fat milk in a buffer containing 140 mmol/L NaCl, 20 mmol/L Tris-HCl (pH 7.5), and 0.1% Tween 20 and incubated with the following primary antibodies: PTG rabbit polyclonal antibody (417,737, MBS, USA), GS rabbit polyclonal antibody (3893, CST, USA), p-GS rabbit polyclonal antibody (3891, CST, USA), GSK3β rabbit polyclonal antibody (9315, CST, USA), p-GSK3β rabbit polyclonal antibody (5558, CST, USA), PYGL rabbit polyclonal antibody (7,603,208, MBS, USA), sterol receptor element-binding protein-1c (SREBP-1c) rabbit polyclonal antibody (sc-366, SANTA CRUZ, USA), acetyl-CoA carboxylase (ACC) rabbit polyclonal antibody (4190S, CST, USA), p-ACC rabbit polyclonal antibody (3661S, CST, USA), 3-hydroxy-3-methylglutaryl-CoA reductase (HMGCR) rabbit polyclonal antibody (sc-33,827, SANTA CRUZ, USA), Histone H3 rabbit polyclonal antibody and β-actin monoclonal mouse antibody (Hua-an Bio-tech Inc., Shanghai, China). Finally, the membranes were incubated with a horseradish peroxidase (HRP) conjugated secondary antibody accordingly for 1 h. The membranes were exposed and visualized using the ECL immobilon western chemiluminescent HRP substrate (WBKLS0500, Millipore, USA). Quantitative analysis was performed using Quantity One software (Bio-Rad Laboratories).

### Assay of glycogen phosphorylase (GPa)

The liver GPa was measured using commercial kits (Solarbio tech, beijing, China). In brief, 190 μl reagent mix composed of 50 mM phosphoglucomutase, 75 mM glucose 6-phosphate dehydrogenase, 0.8% glycogen was pipetted into glass tube at 37 °C for 5 min. 50 mg frozen liver was lysed in 0.9% sodium chloride solution and centrifuged for 10 min at 1000 g. 10 μl supernatant was added to the tube and mixed immediately. Read the absorbance at 340 nm in 5 min and 10 min, respectively. The results were expressed in units of disintegrations per minute per gram tissue. GPa activity was defined by the NADPH production.

### Assays of liver glycogen and muscle glycogen

Liver and muscle glycogen were measured using commercial kits (Meilian tech, shanghai, China). In brief, approximately 25 mg of frozen tissue was lysed in 0.9% sodium chloride solution and centrifuged for 10 min at 1000 g. The supernatant was added to test tubes, along with the addition of 50 μl biotin labeled TXA2 antibody. Heated at 37 °C for 1 h, and discarded the supernatant. Then added 80 μl HRP conjugated secondary antibody and incubated the tube at 37 °C for 30 min. 100 μl substrate was added and incubated at 37 °C for 10 min, followed by the addition of 50 μl stop buffer. Finally read the tube at 450 nm in SpectroMax Plus microplate reader.

### Statistical analysis

The results were expressed as the mean ± SEM. Statistical analysis was performed using one-way analysis of variance (ANOVA) followed by Turkey’s test. *P* < 0.05 was considered to be statistically significant.

## Results

### LGZG decoction alleviated hepatic steatosis and dyslipidemia in NAFLD rats

**Fig. 1:**
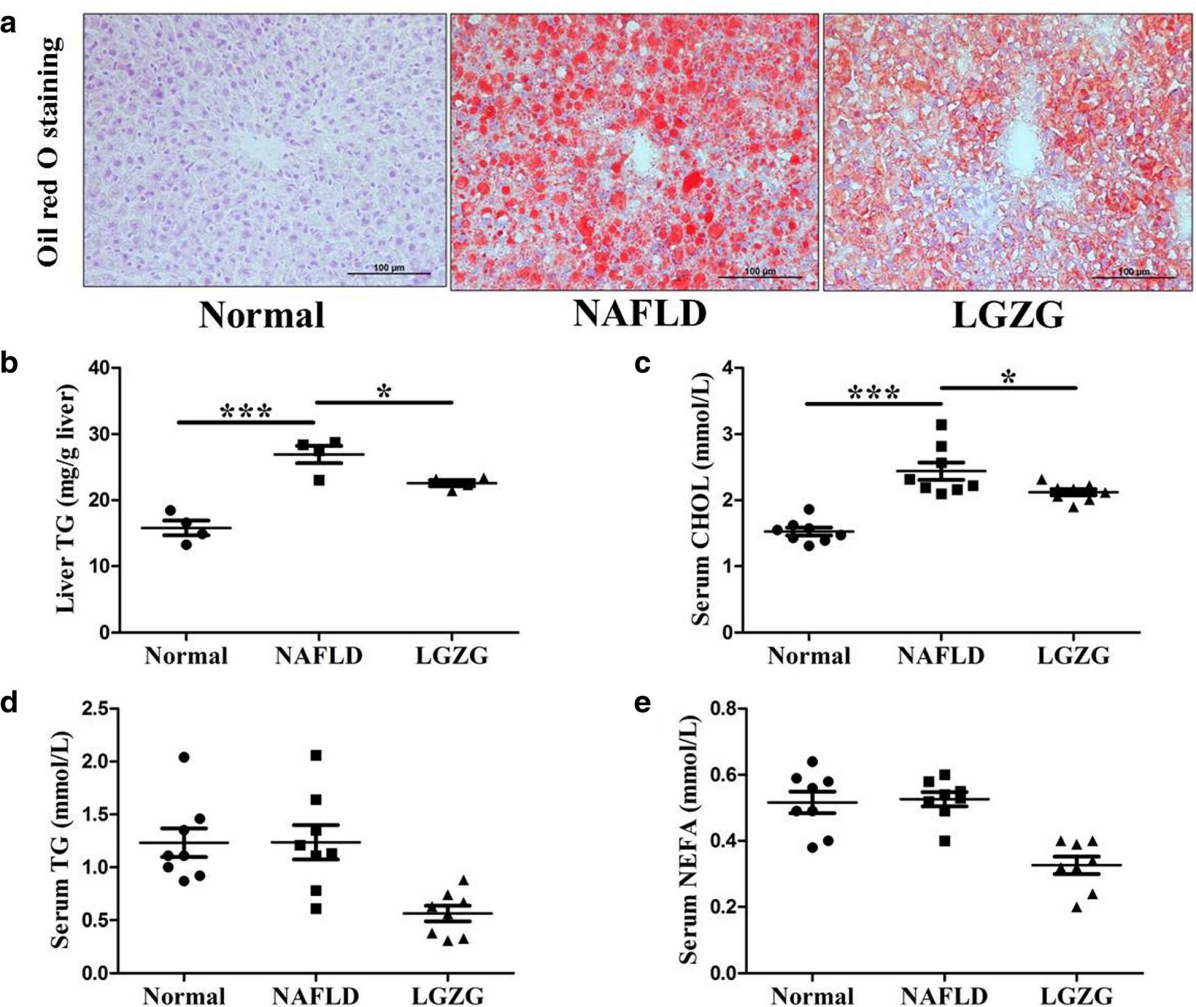
LGZG decoction alleviated hepatic steatosis and dyslipidemia in NAFLD rats. Liver sections were stained with oil Red O (**a**) (image magnification × 200), (**b**) liver TG content was detected (*n* = 4 per group), (**c**) Serum CHOL, (**d**) TG, (**e**) NEFA were analyzed (*n* = 8 per group). Data were presented as means ± SEM. ^*^P < 0.05, ^***^P < 0.001

HFD induced phenotypic characteristics of NAFLD, with obvious hepatic steatosis as evidenced by Oil Red O staining (Fig. 1a), elevated liver TG content (Fig. 1b), and increased serum CHOL (Fig. 1c). 4-week LGZG intervention significantly alleviated hepatic steatosis (Fig. 1a) and decreased liver TG content (Fig. 1b). Analysis of blood lipids showed that LGZG markedly reduced serum CHOL (Fig. 1c). However, serum TG (Fig. 1d) and NEFA (Fig. 1e) showed no significantly difference between control group and NAFLD group.

### PPP1R3C was the target of LGZG decoction in NAFLD rats

**Fig. 2:**
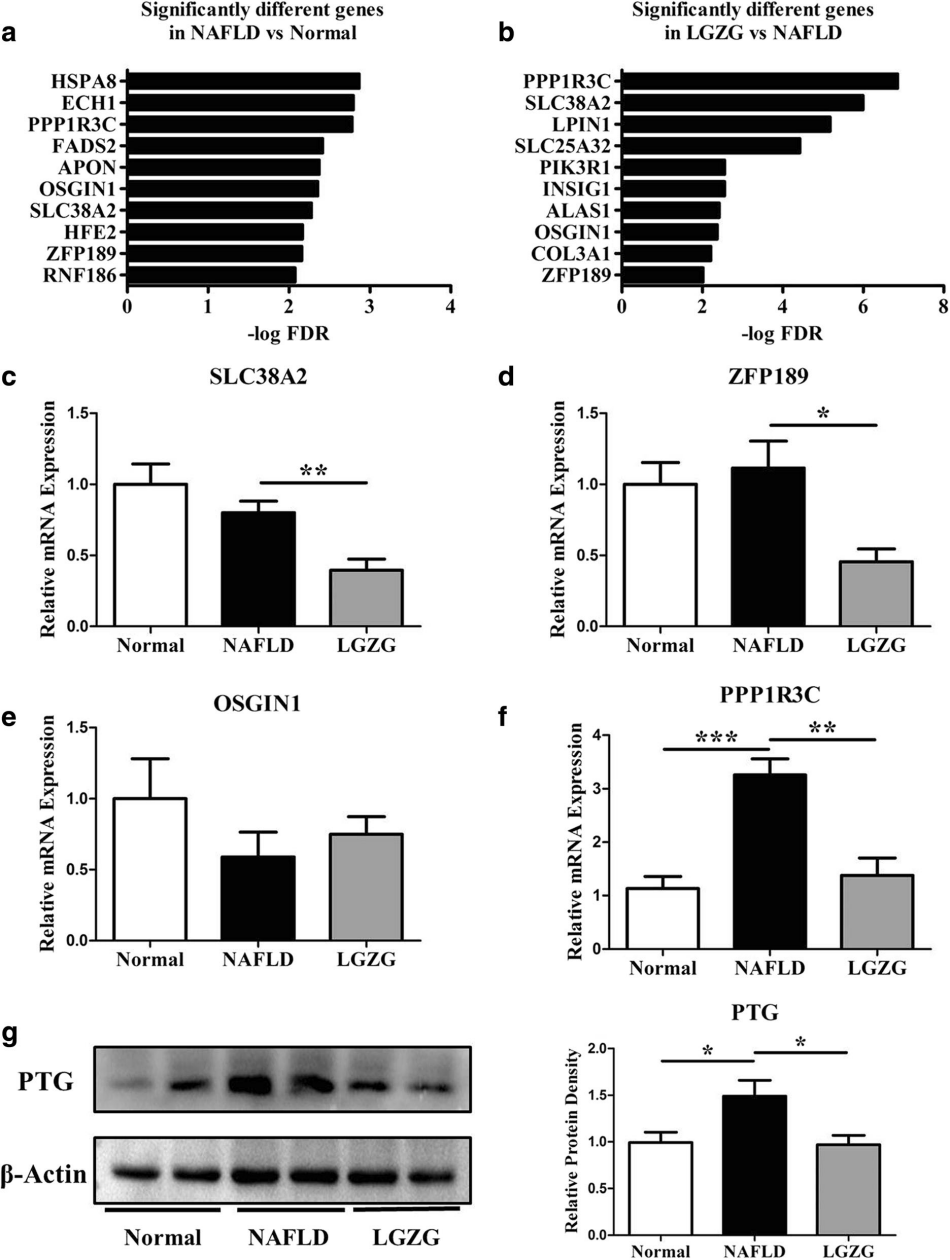
PPP1R3C was the target of LGZG decoction in NAFLD rats. RNA-Sequence identified (**a**) significantly different genes between NAFLD vs Normal group, (**b**) significantly different genes between LGZG vs NAFLD group. RT-qPCR further verified, (**c**) SLC38A2 mRNA expression, (**d**) ZFP189 mRNA expression, (**e**) OSGIN1 mRNA expression, and (**f**) PPP1R3C mRNA expression. Western blot detected (**g**) PTG protein expression. Data were presented as means ± SEM (*n* = 8 per group). *P < 0.05, **P < 0.01, ***P < 0.001

To clarify the mechanisms underlie the efficacy, we performed RNA-sequence on liver tissue from the Normal-, NAFLD- and LGZG-treated rats. Among the differentially expressed mRNAs, we selected the top 10 significantly different mRNAs between Normal and NAFLD groups, and another top 10 significantly different mRNAs between LGZG and NAFLD groups according to false discovery rate (FDR) < 0.01 (Fig. 2a-b, Additional file 2: Table S1). Four overlapping genes, including PPP1R3C, solute carrier family 38 member 2 (SLC38A2), oxidative stress induced growth inhibitor 1 (OSGIN1), zinc finger protein 189 (ZFP189) were found. To verify the expression, we conducted RT-qPCR, although the mRNA expression of SLC38A2 (Fig. 2c) and ZFP189 (Fig. 2d) was significantly decreased in LGZG group compared to NAFLD group, no difference was found between Normal and NAFLD group. Whereas OSGIN1 (Fig. 2e) mRNA expression did not show any difference among groups. Only PPP1R3C mRNA expression was consistent to the RNA-sequence data, showing significantly increase in NAFLD rats compared to Normal rats, while markedly decrease upon LGZG intervention (Fig. 2f). To further verify the expression, we detected the PPP1R3C coded protein expression with western blot, results showed that PTG significantly increased in NAFLD group in comparison to Normal group, while the protein expression markedly decreased by LGZG treatment (Fig. 2g), indicating PPP1R3C was a potential target of LGZG decoction in improving NAFLD.

### LGZG regulated key molecules of glycogen metabolism in NAFLD rats

**Fig. 3:**
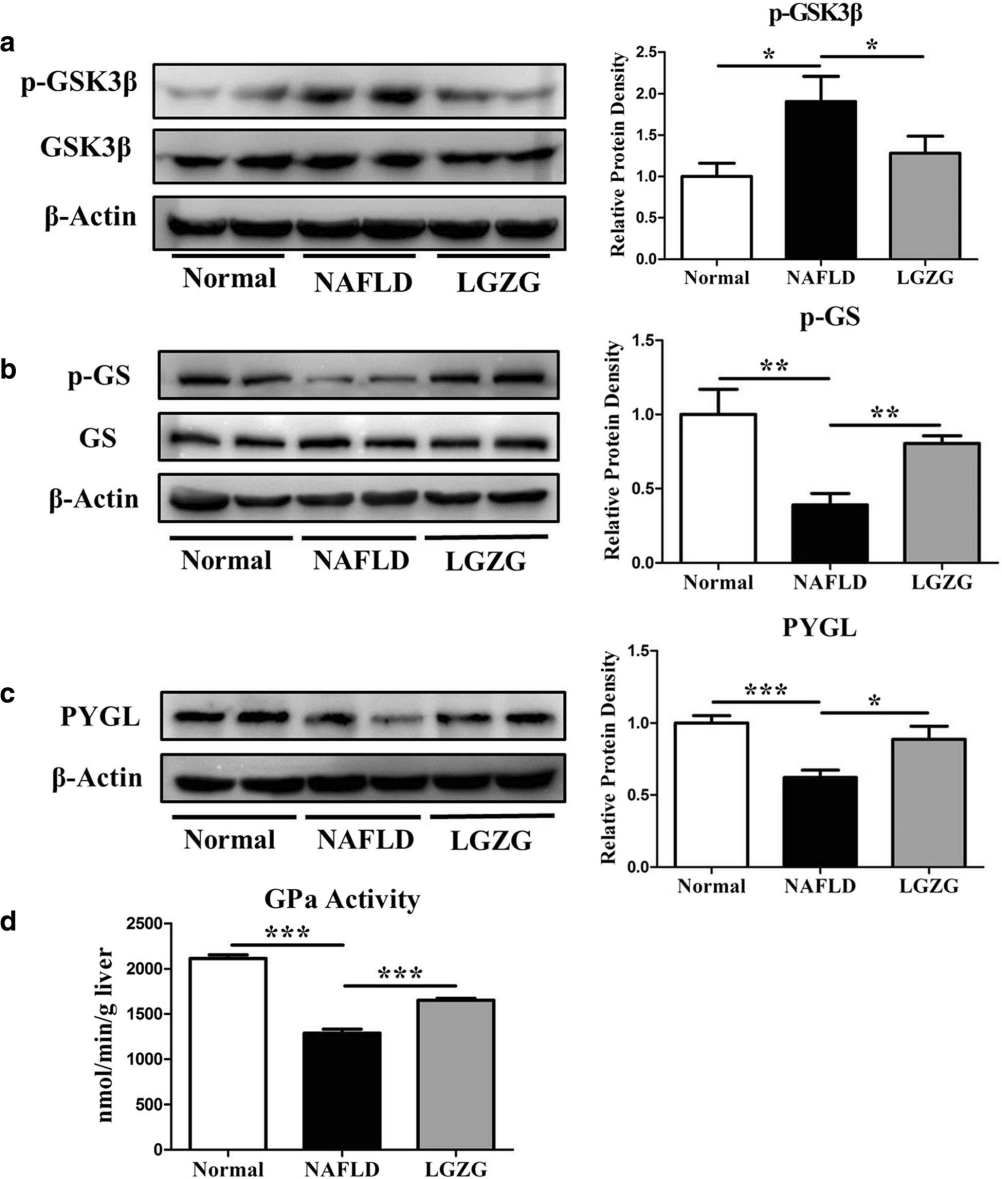
LGZG regulated key molecules of Glycogen metabolism in NAFLD rats. Western blot detected (**a**) p-GSK3β and GSK3βprotein expression, (**b**) p-GS and GS protein expression, (**c**) PYGL protein expression, (**d**) GPa activity was analyzed by ELISA. Data were presented as means ± SEM (*n* = 8 per group). ^*^P < 0.05, ^**^P < 0.01, ^***^P < 0.001

PTG is a scaffolding protein that targets protein phosphatase 1α (PP1α) to glycogen, and plays a vital role in glycogen metabolism. PP1 participates in the regulation of a wide variety of cellular functions by reversible protein phosphorylation, PTG, one of the regulatory subunits of PP1 that localized to glycogen particles are involved in regulating the dephosphorylation of glycogen synthase and phosphorylase. Enzymes GS and GSK3 are the targeting proteins of PTG, we examined their phosphorylation levels and showed that the GSK3β phosphorylation was significantly increased in NAFLD rats, and LGZG significantly decreased GSK3β phosphorylation. Since the activity of GSK3β is determined by dephosphorylation [27], these data suggested LGZG could enhance GSK3β activity (Fig. 3a). The GS phosphorylation decreased obviously in NAFLD, and LGZG can significantly promote GS phosphorylation. Likewise, increased GS phosphorylation means reduced activity [28], indicating the decreased hepatic GS activity upon LGZG intervention in rats (Fig. 3b).

Glycogen phosphorylase in the liver is designated as PYGL, which is also targeting PTG and involves in regulating glycogen degradation [29]. We had detected the protein expression of PYGL and found it significantly decreased in NAFLD rats, whereas LGZG intervention could markedly increase PYGL expression (Fig. 3c). In addition, PTG plays an instrumental role in phosphorylase *a* and *b* inter-conversion. GPa serves as a glucose sensor in liver [30]. GPa in its active R state tightly binds to PTG, and prevents phosphatase activity of PTG to maintain its active status. Here we analyzed the GPa activity, and found LGZG could significantly increase GPa activity of the NAFLD rats (Fig. 3d), indicating the enhanced action in accelerating glycogen degradation.

### LGZG decoction reduced hepatic glycogen in NAFLD rats

**Fig. 4:**
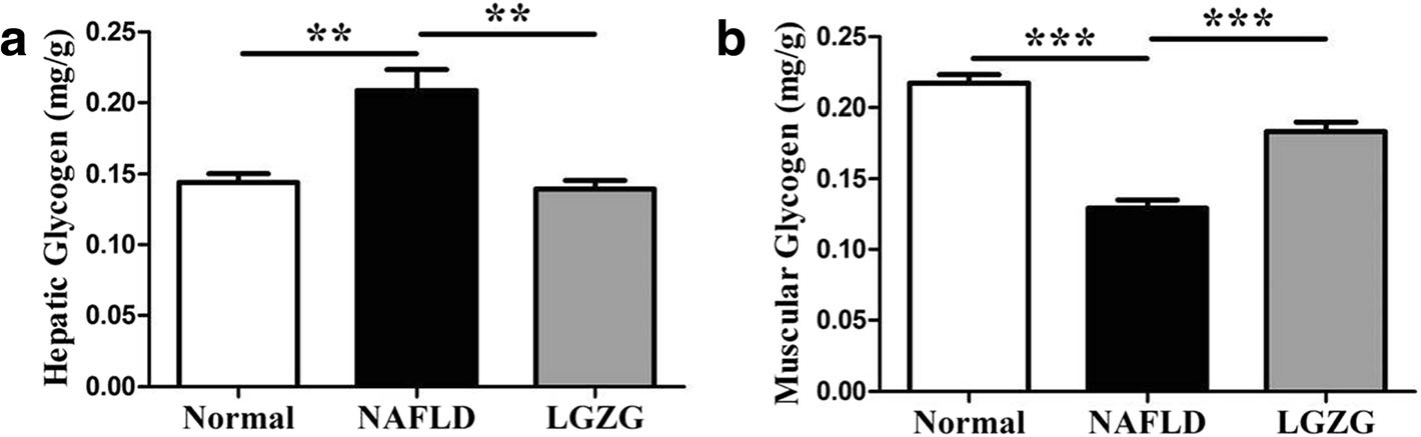
LGZG reduced hepatic glycogen in NAFLD rats. **a** The level of hepatic glycogen, (**b**) The level of muscle glycogen. Data were presented as means ± SEM (*n* = 8 per group). **P < 0.01, ***P < 0.001

PTG and its targeting proteins in the liver contribute to glycogen metabolism, so we conducted experiment to analyze the hepatic glycogen. Our data showed that hepatic glycogen content was significantly increased in NAFLD rats, whereas LGZG intervention markedly decreased hepatic glycogen (Fig. 4a, Table 2), which was consistent to the alteration of PTG and targeting proteins previously. Furthermore, we also found that LGZG increased muscle glycogen in NAFLD rats (Fig. 4b, Table 2).

**Table 2:** Ling-gui-zhu-gan decoction reduces hepatic glycogen (mean ± SEM)

Group	Normal (mg/g)	NAFLD (mg/g)	LGZG (mg/g)
Hepatic Glycogen	0.1440 ± 0.006272	0.2087 ± 0.01469**	0.1395 ± 0.005948^##^
Muscle Glycogen	0.2171 ± 0.006084	0.1292 ± 0.005895**	0.1834 ± 0.006725^##^

*n* = 8 per group, **P < 0.01 vs. Normal group; ^##^*P* < 0.01 vs. NAFLD group

### LGZG decoction inhibited hepatic lipogenesis and cholesterol synthesis molecules

**Fig. 5:**
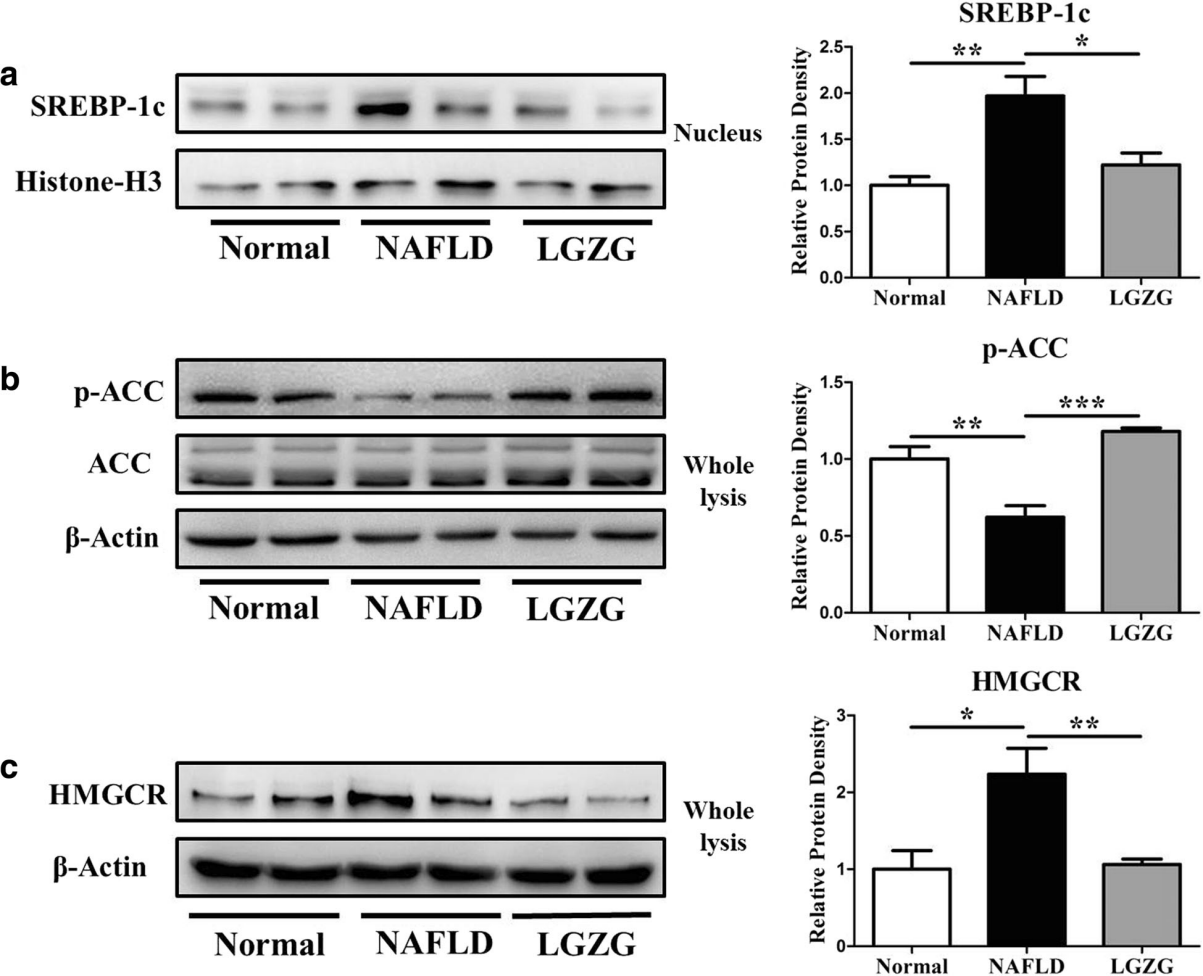
LGZG inhibited hepatic de novo lipogenesis and cholesterol synthesis. **a** SREBP-1c protein expression in nucleus. **b** p-ACC and ACC protein expression. **c** HMGCR protein expression. Data were presented as means ± SEM (*n* = 8 per group). *P < 0.05, **P < 0.01, ***P < 0.001

In the liver, glycogen storage is a transitional action in regulating glucose metabolism, however, accumulated glycogen may also affect hepatic de novo lipogenesis and cholesterol synthesis. To verify the role of LGZG on hepatic de novo lipogenesis, we detected SREBP-1c, the master molecule that responsible for de novo lipogenesis, and found that SREBP-1c protein expression in nucleus significantly increased in NAFLD rats compared to Normal rats, and LGZG intervention significantly decreased the nucleic level of SREBP-1c (Fig. 5a). ACC is the downstream target of SREBP-1c, and its phosphorylation could decrease its activity. Our results showed that LGZG restored the decreased ACC phosphorylation in NAFLD rats (Fig. 5b), indicating the role in inhibiting lipogenesis. In addition, we analyzed the role of LGZG on cholesterol synthesis by evaluating the expression of HMGCR, and identified the suppressing effect of LGZG on HMGCR in NAFLD rats (Fig. 5c). These results indicated that LGZG also inhibited hepatic de novo lipogenesis and cholesterol synthesis, thus contributing its role in preventing NAFLD development.

## Discussion

NAFLD becomes a major public health concern worldwide [7], but the pathological mechanisms are still unclear. Available Pharmaceutical strategies are limited, although several agents are under Phase II or III clinical trials [31–35]. In addition to the effect, safety is also a facet that should not be ignored since long-term intervention might be needed in NAFLD prevention and treatment. LGZG is a classical formula that has been practiced for thousands years in China, recently studies have found its benefit on treating [24, 25].

**Fig. 6:**
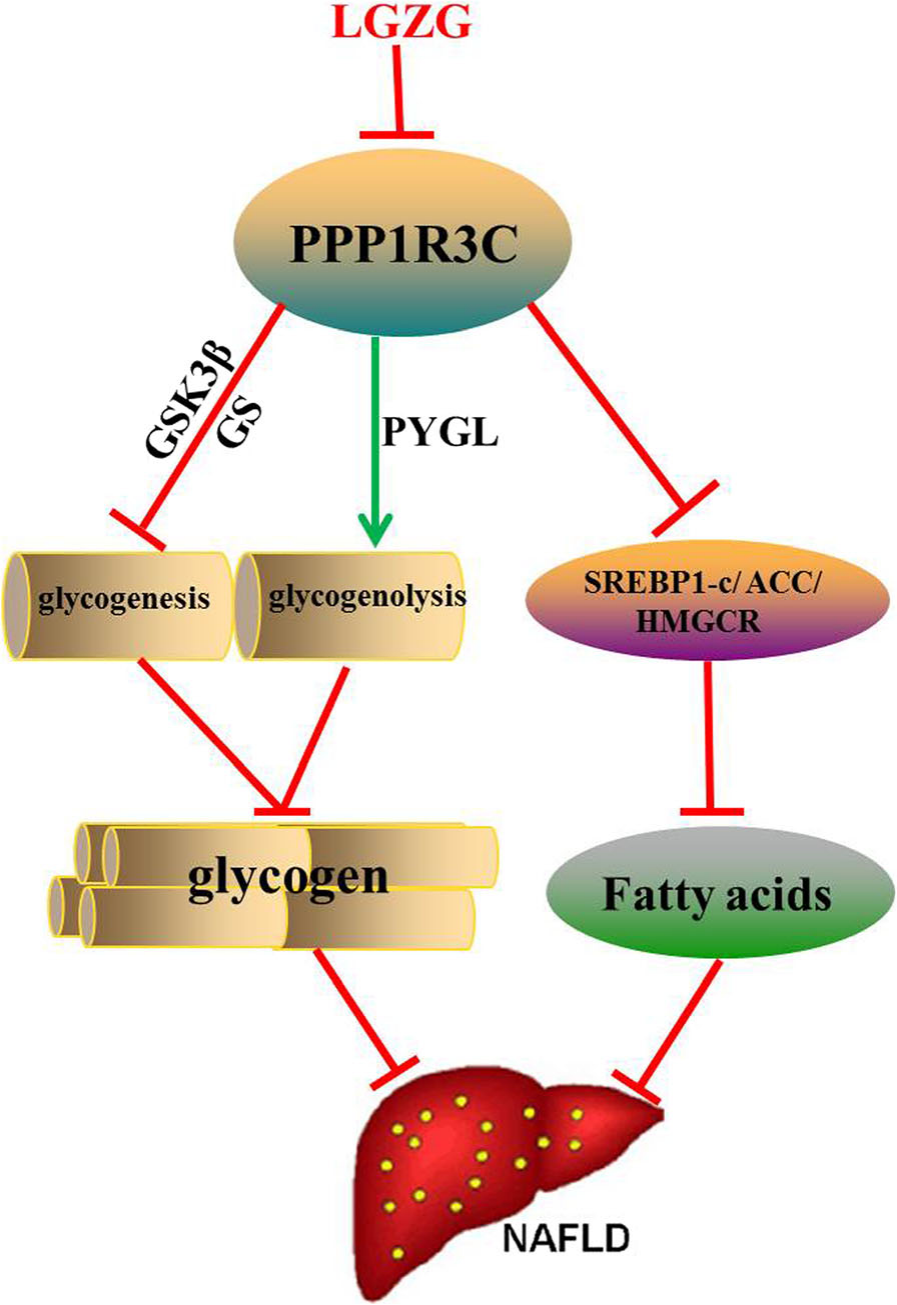
Summary of the study. Traditional Chinese formulae LGZG inhibited PPP1R3C expression. Low PPP1R3C expression could reduce GS activity, promote PYGL expression, and reduce glycogen storage via inhibiting glycogenesis and accelerating glycogenolysis. In addition, low PPP1R3C expression decreased the nucleic SREBP-1c and the ACC activity, which might induce suppressed lipogenesis in the liver. Thus by inhibiting PPP1R3C mediated molecules, LGZG alleviated NAFLD and related complications

Our study applied HFD induced NAFLD rats, confirmed the effect, and uncovered PPP1R3C was responding to LGZG intervention, suggesting PPP1R3C be a possible target of LGZG in preventing NAFLD (Fig. 6). PPP1R3C plays a critical role in glycogen and lipid metabolism, and we observed that LGZG reduced PPP1R3C expression, promoted the activity of PYGL, inhibited the activity of GS in NAFLD rats, resulting in glycogenolysis increase and glycogen synthesis decrease in the liver. Hepatic glycogen content was consistently reduced upon LGZG intervention. In addition, we also detected inhibiting role of LGZG in hepatic de novo lipogenesis and cholesterol synthesis.

PPP1R3C coding protein PTG can mediate glycogen metabolism by regulating the activity of GS and PYGL [17, 36]. PTG enhances the de-phosphorylation of GS and causes the activation of glycogen synthesis [16–18]. Furthermore, PTG is reported to inhibit PYGL expression and GPa de-phosphorylation [16, 17]. Our results also indicated that PTG expression was increased in NAFLD rats, and phosphorylation of GS was decreased, enhancing the activity of GS, however expression and activity of PYGL was decreased. LGZG can inhibit PTG expression, reducing activity of GS, enhancing the activity of PYGL, finally accelerating glycogen metabolism. The result of hepatic glycogen was consistent with enzyme activity, indicating that HFD may promote glycogen accumulation, as a form of excess energy [36].

It is reported that mice that overexpressed PTG presented higher liver glycogen content, but normal or lower liver triglyceride (TG) content [37, 38]. Heterozygous deletion of PPP1R3C in mice showed decreased GS activity and glycogen synthesis rate, but insulin resistance and increased muscle TG content, indicating possible balance between lipid and glycogen. In addition, PTG may regulate SREBP-1c expression [36], while SREBP-1c up-regulation can induce hepatic de novo lipogenesis and SREBP-1c knockdown reduces lipogenesis [39, 40]. Our data indicated inhibiting role of LGZG on lipogenesis also supported the previous finding that LGZG improved steatosis oxidative stress in NAFLD [21, 24].

## Conclusions

In summary, our study highlighted the property of LGZG on regulating PTG, and its beneficial roles on glycogen and lipid metabolism in NAFLD rats. Furthermore, LGZG regulated the molecules in glycogen and lipid synthesis by inhibiting PTG (Fig. 6). Our study provided evidence for applying LGZG in NAFLD treatment, although PTG was a possible target, we could not exclude other possible regulations. In addition, as the exact regulation on PTG is still elusive, further studies are in need to clarify potential mechanisms.

## Additional files

Additional file 1: **Figure S1.** Fingerprint spectrum of LGZG by LC-MS. 1-Characteristic peak of *Ramulus Cinnamomi*; 2-Characteristic peak of *Rhizoma Atractylodis Macrocephalae*; 3-Characteristic peak of *Radix Glycyrrhizae*; 4-Characteristic peak of *Radix Glycyrrhizae*; 5-Liquiritin; 6-Cinnamic acid; 7-Cinnamic aldehyde; 8-Glycyrrhizic acid; 9-Atractylenolide III; 10-Characteristic peak of *Rhizoma Atractylodis Macrocephalae.* (JPG 62 kb)

Additional file 2: **Table S1.** The overlapped differentially expressed genes. (XLSX 12 kb)

## Abbreviations

ACC: Acetyl-CoA carboxylase
CHOL: Cholesterol
GPa: Glycogen phosphorylase a
GS: Glycogen synthase
GSK3β: Glycogen synthase kinase 3β
HFD: High fat diet
HMGCR: 3-hydroxy-3-methylglutaryl-CoA reductase
HRP: Horseradish peroxidase
LGZG: Ling-gui-zhu-gan decoction
NAFLD: Non-alcoholic fatty liver disease
NEFA: Non-esterified fatty acid
OSGIN1: Oxidative stress induced growth inhibitor 1
PCR: Polymerase chain reaction
PP1: Protein phosphatase-1
PP1α: Protein phosphatase 1α
PPP1R3C: Protein phosphatase 1 regulatory subunit 3C
PTG: Protein targeting to glycogen
PYGL: Glycogen phosphorylase liver type
SLC38A2: Solute carrier family 38 member 2
SREBP-1c: Sterol receptor element-binding protein-1c
TCM: Traditional Chinese medicine
TG: Triglyceride
ZFP189: Zinc finger protein 189
