# Supplementary figures and images for: The traditional Chinese formulae Ling-gui-zhu-gan decoction alleviated non-alcoholic fatty liver disease via inhibiting PPP1R3C mediated molecules
Source: BMC Complement Altern Med. 2019 Jan 7;19:8. doi: 10.1186/s12906-018-2424-1 (PMC6323852; doi:10.1186/s12906-018-2424-1)

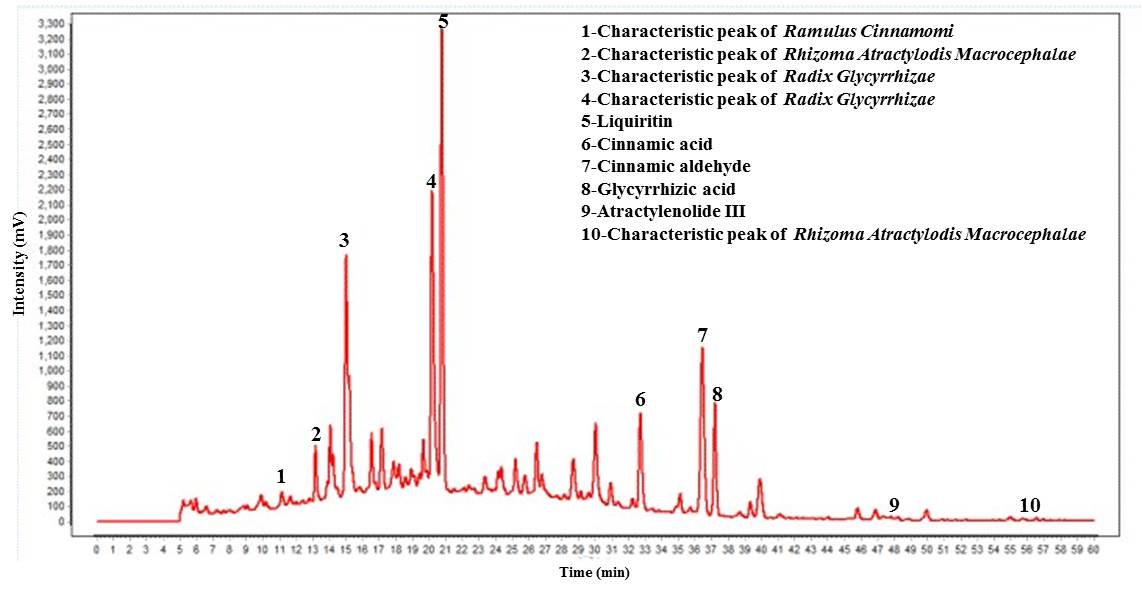

Supplement: Supplementary file 1 — Figure S1. Fingerprint spectrum of LGZG by LC-MS. 1-Characteristic peak of Ramulus Cinnamomi; 2-Characteristic peak of Rhizoma Atractylodis Macrocephalae; 3-Characteristic peak of Radix Glycyrrhizae; 4-Characteristic peak of Radix Glycyrrhizae; 5-Liquiritin; 6-Cinnamic acid; 7-Cinnamic aldehyde; 8-Glycyrrhizic acid; 9-Atractylenolide III; 10-Characteristic peak of Rhizoma Atractylodis Macrocephalae. (JPG 62 kb) [file 12906_2018_2424_MOESM1_ESM.jpg]
